# Potassium sodium (2*R*,3*R*)-tartrate tetra­hydrate: the paraelectric phase of Rochelle salt at 105 K

**DOI:** 10.1107/S1600536808005266

**Published:** 2008-03-05

**Authors:** Carl Henrik Görbitz, Einar Sagstuen

**Affiliations:** a Department of Chemistry, University of Oslo, PO Box 1033 Blindern, N-0315 Oslo, Norway; b Department of Physics, University of Oslo, PO Box 1048 Blindern, N-0316 Oslo, Norway

## Abstract

Rochelle salt, K^+^·Na^+^·C_4_H_4_O_6_^2−^·4H_2_O, is known for its remarkable ferroelectric state between 255 and 297 K. The current investigation, based on data collected at 105 K, provides very accurate structural information for the low-temperature paraelectric form. Unlike the ferroelectric form, there is only one tartrate molecule in the asymmetric unit, and the structure displays no disorder to large anisotropic atomic displacements.

## Related literature

For previous and related structures, see: Beevers & Hughes (1941 ▶); Iwata *et al.* (1989 ▶); Solans *et al.* (1997 ▶); Ottenz *et al.* (1998 ▶); Hinazumi & Mitsui (1972 ▶); Kay (1978 ▶); Kuroda & Mason (1981 ▶); Brożek & Stadnicka (1994 ▶); Suzuki *et al.* (1996*a* ▶,*b* ▶); Ambady & Kartha (1968 ▶); Boese *et al.* (1995 ▶). For irradiation studies, see: Suzuki (1974 ▶); Treeck, van & Windsch (1977 ▶). For a description of the Cambridge Structural Database, see: Allen (2002 ▶).
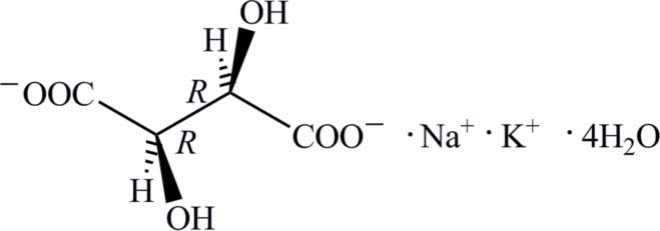


## Experimental

### 

#### Crystal data

K^+^·Na^+^·C_4_H_4_O_6_^2−^·4H_2_O
*M* *_r_* = 282.23
Orthorhombic, 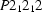

*a* = 11.7859 (6) Å
*b* = 14.1972 (7) Å
*c* = 6.1875 (3) Å
*V* = 1035.33 (9) Å^3^
*Z* = 4
Mo *K*α radiation
μ = 0.60 mm^−1^
*T* = 105 (2) K
0.5 mm (radius)

#### Data collection

Siemens SMART CCD diffractometer
Absorption correction: multi-scan (*SADABS*; Sheldrick, 1996 ▶) *T*_min_ = 0.398, *T*_max_ = 0.551 (expected range = 0.722–1.000)
33523 measured reflections
10040 independent reflections
8947 reflections with *I* > 2σ(*I*)
*R*_int_ = 0.037

#### Refinement

*R*[*F* ^2^ > 2σ(*F* ^2^)] = 0.029
*wR*(*F* ^2^) = 0.069
*S* = 1.06
10040 reflections
195 parameters
12 restraints
All H-atom parameters refined
Δρ_max_ = 0.50 e Å^−3^
Δρ_min_ = −0.73 e Å^−3^
Absolute structure: Flack, 1983 ▶, 4266 Friedel pairs
Flack parameter: 0.044 (14)

### 

Data collection: *SMART* (Bruker, 1998 ▶); cell refinement: *SAINT-Plus* (Bruker, 2001 ▶); data reduction: *SAINT-Plus*; program(s) used to solve structure: *SHELXTL* (Sheldrick, 2008 ▶); program(s) used to refine structure: *SHELXTL*; molecular graphics: *SHELXTL*; software used to prepare material for publication: *SHELXTL*.

## Supplementary Material

Crystal structure: contains datablocks I, global. DOI: 10.1107/S1600536808005266/bg2163sup1.cif

Structure factors: contains datablocks I. DOI: 10.1107/S1600536808005266/bg2163Isup2.hkl

Additional supplementary materials:  crystallographic information; 3D view; checkCIF report

## Figures and Tables

**Table 1:** Hydrogen-bond geometry (Å, °)

*D*—H⋯*A*	*D*—H	H⋯*A*	*D*⋯*A*	*D*—H⋯*A*
O5—H5⋯O2	0.789 (17)	2.031 (16)	2.5946 (6)	128.2 (14)
O6—H6⋯O4*W*^i^	0.861 (16)	1.968 (16)	2.8119 (7)	166.5 (16)
O1*W*—H11*W*⋯O6	0.824 (8)	1.960 (8)	2.7832 (6)	176.8 (15)
O1*W*—H12*W*⋯O4^ii^	0.843 (9)	2.010 (9)	2.8500 (7)	174.8 (18)
O2*W*—H21*W*⋯O3^iii^	0.868 (9)	1.830 (9)	2.6941 (7)	173.4 (19)
O2*W*—H22*W*⋯O2^iv^	0.862 (9)	1.890 (9)	2.7505 (7)	175.5 (19)
O3*W*—H31*W*⋯O6^v^	0.843 (9)	2.391 (15)	3.1029 (7)	142.5 (19)
O3*W*—H31*W*⋯O2^vi^	0.843 (9)	2.499 (17)	3.1181 (7)	131.0 (17)
O3*W*—H31*W*⋯O3^v^	0.843 (9)	2.584 (14)	3.1569 (8)	126.2 (15)
O3*W*—H32*W*⋯O4^vii^	0.862 (8)	1.926 (8)	2.7842 (8)	173.8 (16)
O4*W*—H41*W*⋯O1^viii^	0.858 (9)	1.888 (10)	2.7124 (6)	160.4 (19)
O4*W*—H42*W*⋯O3*W*^iv^	0.836 (8)	1.939 (9)	2.7532 (8)	164.4 (16)

Symmetry codes: (i) 
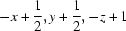
; (ii) 
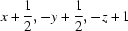
; (iii) 
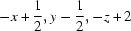
; (iv) 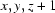; (v) 
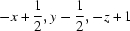
; (vi) 
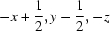
; (vii) 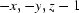; (viii) 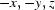.

**Fig. 1.**
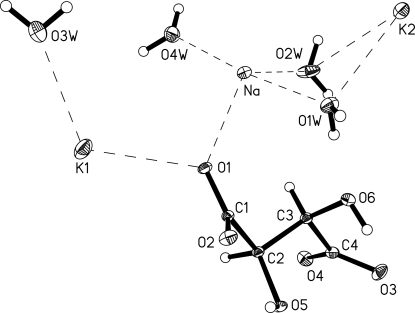
: The molecular structure of (I). Displacement ellipsoids are shown at the 50% probability level. Metal ccordination has been indicated by dashed lines.

**Fig. 2.**
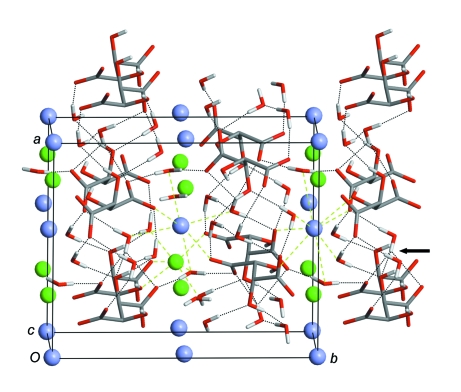
: Crystal packing arrangement viewed approximately along the c axis. H atoms bonded to C have been left out for clarity. Na+ is yellow, K+ is light blue with K1 at the centre of the unit cell and K2 at the cell edge. Hydrogen bonds are shown as black dotted lines while ligand coordination is indicated in orange for three selected metal ions. The arrow points to H31W, which is involved in a four-center hydrogen bond.

## supplementary crystallographic information

### Comment

The radiation-induced free radical chemistry of dicarboxylic acids and their salts has received attention for several decades. The Rochelle salt, is of particular interest as it as it exhibits a ferroelectric phase between 255 and 297 K, where the structure is monoclinic, space group *P*2_1_; outside this temperature range the compound is paraelectric and presents orthorhombic phases in space group *P*2_1_2_1_2. The nature of the radicals formed in Rochelle salt is currently investigated in order to understand the mechanisms producing changes in the ferroelectric properties of this compound upon irradiation (Suzuki, 1974; Treeck, van & Windsch, 1977). For the analysis of the electron magnetic resonance data, precise knowledge of the low-temperature orthorhombic form is necessary. Structural data for the high-temperature orthorhombic form were first provided by Beevers & Hughes (1941). Iwata *et al.* (1989) carried out a neutron diffraction study for both orthorhombic forms; more accurate X-ray diffraction studies were later presented by Solans *et al.* (1997), who concluded that differences between the two *P*2_1_2_1_2 states are "small but significant". None of these structures are, however, available in the Cambridge Structural Database (Version 5.29 of November 2007; Allen, 2002). A high-precision redetermination of Rochelle salt at low temperature has therefore been executed.

The molecular structure of (I) is shown in Fig. 1. The crystal packing arrangement, illustrated in Fig. 2, is very similar to those found in the *P*2_1_2_1_2 structures of other salts of tartaric acid in which Na^+^ is replaced by Li^+^ and/or K^+^ by NH_4_^+^ or Tl^+^ [Li^+^/K^+^: Ottenz *et al.*, 1998; Li^+^/NH_4_^+^: Hinazumi & Mitsui, 1972; Li^+^/Tl^+^: Kay, 1978; Na^+^/NH_4_^+^ (II): Kuroda & Mason, 1981; Brożek & Stadnicka, 1994; Suzuki *et al.*, 1996*a*] as well as in salts where K^+^ has been only partly replaced by NH_4_^+^ (Suzuki *et al.*, 1996*a*; Suzuki *et al.*, 1996*b*). The pure sodium (Ambady & Kartha, 1968) or potassium salts (Boese *et al.*, 1995) on the other hand, have completely different structures.

Hydrogen bonds are listed in Table 1, the most unusual feature is the almost symmetric four-center interaction involving H31W.

When K^+^ is replaced by NH_4_^+^ [as, for instance, in II] the four shortest K2···O contacts are converted into hydrogen bonds, while only the two K1···O4 interactions are transformed into short hydrogen bonds, the K1···O1W and K1···O2W contacts being replaced by a three-center hydrogen bond.

### Experimental

Rochelle salt was obtained from Sigma-Aldrich and tetrahydrate crystals were grown from saturated aqueous solutions. A large block-shaped speciemen was ground into a sphere in a mill and used for data collection.

### Refinement

Full isotropic refinement was carried out for all H atoms.

### Crystal data

**Table:**

K^+^·Na^+^·C_4_H_4_O_6_^2–^·4H_2_O	*D*_x_ = 1.811 Mg m^−^^3^
*M**_r_* = 282.23	Mo *K*α radiation λ = 0.71073 Å
Orthorhombic, *P*2_1_2_1_2	Cell parameters from 10000 reflections
*a* = 11.7859 (6) Å	θ = 2.9–49.7º
*b* = 14.1972 (7) Å	µ = 0.60 mm^−^^1^
*c* = 6.1875 (3) Å	*T* = 105 (2) K
*V* = 1035.33 (9) Å^3^	Sphere, colourless
*Z* = 4	0.5 mm (radius)
*F*_000_ = 584	

### Data collection

**Table:**

Siemens SMART CCD diffractometer	10040 independent reflections
Radiation source: fine-focus sealed tube	8947 reflections with *I* > 2σ(*I*)
Monochromator: graphite	*R*_int_ = 0.037
Detector resolution: 8.3 pixels mm^-1^	θ_max_ = 49.7º
*T* = 105(2) K	θ_min_ = 2.9º
sets of exposures each taken over 0.3° ω rotation scans	*h* = −25→25
Absorption correction: multi-scan(SADABS; Sheldrick, 1996)	*k* = −28→30
*T*_min_ = 0.398, *T*_max_ = 0.551	*l* = −12→12
33523 measured reflections	

### Refinement

**Table:**

Refinement on *F*^2^	All H-atom parameters refined
Least-squares matrix: full	*w* = 1/[σ^2^(*F*_o_^2^) + (0.0324*P*)^2^ + 0.0088*P*] where *P* = (*F*_o_^2^ + 2*F*_c_^2^)/3
*R*[*F*^2^ > 2σ(*F*^2^)] = 0.029	(Δ/σ)_max_ = 0.002
*wR*(*F*^2^) = 0.069	Δρ_max_ = 0.50 e Å^−^^3^
*S* = 1.06	Δρ_min_ = −0.73 e Å^−^^3^
10040 reflections	Extinction correction: SHELXTL (Bruker, 2000), Fc^*^=kFc[1+0.001xFc^2^λ^3^/sin(2θ)]^-1/4^
195 parameters	Extinction coefficient: 0.132 (3)
12 restraints	Absolute structure: Flack, 1983, 4266 Friedel pairs
Primary atom site location: structure-invariant direct methods	Flack parameter: 0.044 (14)
Hydrogen site location: difference Fourier map	

### Special details

**Table:**

Geometry. All e.s.d.'s (except the e.s.d. in the dihedral angle between two l.s. planes) are estimated using the full covariance matrix. The cell e.s.d.'s are taken into account individually in the estimation of e.s.d.'s in distances, angles and torsion angles; correlations between e.s.d.'s in cell parameters are only used when they are defined by crystal symmetry. An approximate (isotropic) treatment of cell e.s.d.'s is used for estimating e.s.d.'s involving l.s. planes.
Refinement. Data were collected by measuring six sets of exposures with the detector set at 2θ = 29° and 65°, crystal-to-detector distance 5.00 cm. Refinement of *F*^2^ against ALL reflections.

### Fractional atomic coordinates and isotropic or equivalent isotropic displacement parameters (Å^2^)

**Table:**

	*x*	*y*	*z*	*U*_iso_*/*U*_eq_	
K1	0.0000	0.0000	0.04255 (4)	0.02054 (4)	
K2	0.5000	0.0000	0.83902 (3)	0.01318 (3)	
Na	0.23248 (2)	−0.007143 (18)	0.51526 (4)	0.01041 (4)	
O1	0.12000 (3)	0.10859 (3)	0.34799 (7)	0.01016 (5)	
O2	0.21269 (4)	0.20379 (3)	0.11755 (7)	0.01211 (6)	
O3	0.22830 (4)	0.40729 (3)	0.82011 (8)	0.01585 (7)	
O4	0.04765 (4)	0.35891 (3)	0.84893 (8)	0.01439 (7)	
O5	0.16547 (4)	0.35790 (3)	0.32421 (7)	0.01060 (5)	
H5	0.1932 (12)	0.3393 (11)	0.216 (3)	0.021 (3)*	
O6	0.29638 (3)	0.24888 (3)	0.63394 (7)	0.01132 (6)	
H6	0.3284 (13)	0.2989 (11)	0.584 (3)	0.025 (3)*	
C1	0.15538 (4)	0.18798 (3)	0.28320 (8)	0.00798 (6)	
C2	0.12505 (4)	0.27375 (3)	0.42269 (8)	0.00802 (6)	
H2	0.0351 (13)	0.2714 (11)	0.429 (3)	0.024 (3)*	
C3	0.17752 (4)	0.26353 (3)	0.64784 (8)	0.00867 (6)	
H3	0.1368 (13)	0.2117 (11)	0.726 (3)	0.027 (4)*	
C4	0.14865 (5)	0.35032 (4)	0.78496 (9)	0.01035 (6)	
O1W	0.39615 (4)	0.08350 (3)	0.48487 (8)	0.01405 (7)	
H11W	0.3642 (12)	0.1317 (8)	0.527 (2)	0.023 (3)*	
H12W	0.4433 (14)	0.0974 (13)	0.388 (3)	0.058 (6)*	
O2W	0.23689 (6)	0.04149 (3)	0.87925 (8)	0.02083 (10)	
H21W	0.2524 (16)	0.0009 (10)	0.980 (2)	0.045 (5)*	
H22W	0.2331 (16)	0.0930 (8)	0.953 (3)	0.044 (5)*	
O3W	0.05896 (4)	−0.19201 (4)	−0.03036 (10)	0.01860 (8)	
H31W	0.1210 (11)	−0.2072 (13)	0.028 (3)	0.051 (6)*	
H32W	0.0307 (13)	−0.2458 (8)	−0.066 (3)	0.037 (5)*	
O4W	0.07835 (4)	−0.10799 (4)	0.57031 (9)	0.01684 (8)	
H41W	0.0099 (9)	−0.1009 (14)	0.526 (3)	0.054 (6)*	
H42W	0.0734 (12)	−0.1431 (10)	0.6784 (19)	0.026 (4)*	

### Atomic displacement parameters (Å^2^)

**Table:**

	*U*^11^	*U*^22^	*U*^33^	*U*^12^	*U*^13^	*U*^23^
K1	0.02786 (8)	0.01565 (7)	0.01810 (8)	−0.00967 (7)	0.000	0.000
K2	0.01563 (5)	0.01227 (5)	0.01164 (6)	−0.00229 (5)	0.000	0.000
Na	0.01174 (8)	0.00770 (8)	0.01179 (9)	−0.00031 (6)	−0.00041 (6)	0.00065 (7)
O1	0.01294 (12)	0.00587 (11)	0.01166 (15)	−0.00070 (9)	−0.00034 (11)	0.00031 (10)
O2	0.01710 (14)	0.00966 (13)	0.00956 (15)	−0.00019 (11)	0.00367 (11)	−0.00079 (10)
O3	0.02292 (18)	0.01090 (15)	0.01373 (18)	−0.00454 (13)	−0.00006 (14)	−0.00398 (12)
O4	0.01600 (14)	0.01549 (16)	0.01168 (16)	0.00482 (12)	0.00046 (12)	−0.00285 (13)
O5	0.01694 (14)	0.00605 (12)	0.00880 (14)	−0.00054 (10)	−0.00024 (11)	0.00062 (9)
O6	0.01094 (12)	0.00955 (13)	0.01345 (16)	0.00182 (10)	−0.00145 (10)	0.00072 (11)
C1	0.01007 (13)	0.00605 (13)	0.00782 (15)	0.00050 (11)	−0.00097 (11)	−0.00050 (10)
C2	0.01034 (13)	0.00591 (13)	0.00780 (16)	0.00042 (11)	−0.00044 (11)	−0.00038 (10)
C3	0.01152 (13)	0.00669 (14)	0.00780 (16)	0.00042 (11)	−0.00063 (12)	−0.00042 (11)
C4	0.01571 (16)	0.00818 (15)	0.00715 (16)	0.00110 (13)	−0.00097 (13)	−0.00082 (11)
O1W	0.01351 (14)	0.01088 (14)	0.01774 (19)	−0.00051 (11)	0.00272 (12)	0.00068 (12)
O2W	0.0434 (3)	0.00952 (15)	0.00958 (18)	0.00494 (17)	0.00155 (17)	−0.00014 (11)
O3W	0.01445 (15)	0.0230 (2)	0.0183 (2)	0.00034 (14)	−0.00277 (14)	0.00046 (16)
O4W	0.01251 (14)	0.01583 (17)	0.0222 (2)	−0.00316 (12)	−0.00284 (13)	0.00420 (15)

### Geometric parameters (Å, °)

**Table:**

K1—O1	2.8194 (4)	O2—C1	1.2479 (6)
K1—O1^i^	2.8194 (4)	O3—C4	1.2581 (7)
K1—O3W^i^	2.8491 (6)	O4—C4	1.2604 (7)
K1—O3W	2.8491 (6)	O5—C2	1.4232 (6)
K1—O2W^ii^	3.0271 (7)	O5—H5	0.789 (17)
K1—O2W^iii^	3.0270 (7)	O6—C3	1.4188 (6)
K2—O1W	2.7758 (5)	O6—H6	0.861 (16)
K2—O1W^iv^	2.7758 (5)	C1—C2	1.5348 (7)
K2—O4^v^	2.8383 (5)	C2—C3	1.5311 (7)
K2—O4^vi^	2.8383 (5)	C2—H2	1.062 (15)
K2—O5^vii^	2.9822 (4)	C3—C4	1.5342 (7)
K2—O5^viii^	2.9822 (4)	C3—H3	1.004 (17)
K2—O2W	3.1662 (7)	O1W—H11W	0.824 (8)
K2—O2W^iv^	3.1662 (7)	O1W—H12W	0.843 (9)
Na—O1W	2.3264 (5)	O2W—H21W	0.868 (9)
Na—O4W	2.3379 (5)	O2W—H22W	0.862 (9)
Na—O1	2.3512 (5)	O3W—H31W	0.843 (9)
Na—O2W	2.3562 (6)	O3W—H32W	0.862 (8)
Na—O3^vii^	2.4485 (6)	O4W—H41W	0.858 (9)
Na—O5^vii^	2.4707 (5)	O4W—H42W	0.836 (8)
O1—C1	1.2668 (6)		
			
O2—C1—O1	126.74 (5)	C2—C3—C4	109.72 (4)
O2—C1—C2	116.44 (4)	O6—C3—H3	113.2 (9)
O1—C1—C2	116.82 (4)	C2—C3—H3	108.4 (10)
O5—C2—C3	109.50 (4)	C4—C3—H3	102.5 (10)
O5—C2—C1	110.32 (4)	O3—C4—O4	126.03 (5)
C3—C2—C1	110.02 (4)	O3—C4—C3	116.52 (5)
O5—C2—H2	112.1 (8)	O4—C4—C3	117.44 (5)
C3—C2—H2	111.5 (9)	H11W—O1W—H12W	109.6 (12)
C1—C2—H2	103.2 (9)	H21W—O2W—H22W	101.2 (11)
O6—C3—C2	110.95 (4)	H31W—O3W—H32W	102.7 (11)
O6—C3—C4	111.73 (4)	H41W—O4W—H42W	105.2 (11)
			
O2—C1—C2—O5	3.05 (6)	O5—C2—C3—C4	57.57 (5)
O1—C1—C2—O5	−177.09 (4)	C1—C2—C3—C4	178.98 (4)
O2—C1—C2—C3	−117.87 (5)	O6—C3—C4—O3	16.43 (7)
O1—C1—C2—C3	61.98 (5)	C2—C3—C4—O3	−107.06 (5)
O5—C2—C3—O6	−66.37 (5)	O6—C3—C4—O4	−164.68 (5)
C1—C2—C3—O6	55.04 (5)	C2—C3—C4—O4	71.84 (6)

Symmetry codes: (i) −*x*, −*y*, *z*; (ii) −*x*, −*y*, *z*−1; (iii) *x*, *y*, *z*−1; (iv) −*x*+1, −*y*, *z*; (v) *x*+1/2, −*y*+1/2, −*z*+2; (vi) −*x*+1/2, *y*−1/2, −*z*+2; (vii) −*x*+1/2, *y*−1/2, −*z*+1; (viii) *x*+1/2, −*y*+1/2, −*z*+1.

### Hydrogen-bond geometry (Å, °)

**Table:**

*D*—H···*A*	*D*—H	H···*A*	*D*···*A*	*D*—H···*A*
O5—H5···O2	0.789 (17)	2.031 (16)	2.5946 (6)	128.2 (14)
O6—H6···O4W^ix^	0.861 (16)	1.968 (16)	2.8119 (7)	166.5 (16)
O1W—H11W···O6	0.824 (8)	1.960 (8)	2.7832 (6)	176.8 (15)
O1W—H12W···O4^viii^	0.843 (9)	2.010 (9)	2.8500 (7)	174.8 (18)
O2W—H21W···O3^vi^	0.868 (9)	1.830 (9)	2.6941 (7)	173.4 (19)
O2W—H22W···O2^x^	0.862 (9)	1.890 (9)	2.7505 (7)	175.5 (19)
O3W—H31W···O6^vii^	0.843 (9)	2.391 (15)	3.1029 (7)	142.5 (19)
O3W—H31W···O2^xi^	0.843 (9)	2.499 (17)	3.1181 (7)	131.0 (17)
O3W—H31W···O3^vii^	0.843 (9)	2.584 (14)	3.1569 (8)	126.2 (15)
O3W—H32W···O4^ii^	0.862 (8)	1.926 (8)	2.7842 (8)	173.8 (16)
O4W—H41W···O1^i^	0.858 (9)	1.888 (10)	2.7124 (6)	160.4 (19)
O4W—H42W···O3W^x^	0.836 (8)	1.939 (9)	2.7532 (8)	164.4 (16)

Symmetry codes: (ix) −*x*+1/2, *y*+1/2, −*z*+1; (viii) *x*+1/2, −*y*+1/2, −*z*+1; (vi) −*x*+1/2, *y*−1/2, −*z*+2; (x) *x*, *y*, *z*+1; (vii) −*x*+1/2, *y*−1/2, −*z*+1; (xi) −*x*+1/2, *y*−1/2, −*z*; (ii) −*x*, −*y*, *z*−1; (i) −*x*, −*y*, *z*.
